# Takotsubo Cardiomyopathy in a 66-Year-Old Woman: A Case of Stress-Induced Cardiomyopathy Mimicking Acute Coronary Syndrome in the Presence of Cardiovascular Risk Factors

**DOI:** 10.7759/cureus.76909

**Published:** 2025-01-04

**Authors:** Jesse O'Rorke, Greyson Butler, Ramesh Chandra

**Affiliations:** 1 Medicine, Lee Health, Fort Myers, USA; 2 Medicine, Lake Erie College of Osteopathic Medicine, Bradenton, USA; 3 Medicine, Lake Erie College of Osteopathic Medicine, Lakewood Ranch, USA; 4 Interventional Cardiology, Lee Health, Fort Myers, USA

**Keywords:** acute coronary syndrome, broken heart syndrome, heart failure with reduced ejection fraction, left ventriculography, takotsubo cardiomyopathy

## Abstract

Takotsubo cardiomyopathy (TC), also known as stress-induced cardiomyopathy or "broken heart syndrome," is a transient cardiac syndrome characterized by acute left ventricular dysfunction, often mimicking acute coronary syndrome (ACS). TC is triggered by emotional or physical stress and presents with chest pain, electrocardiographic abnormalities, and elevated cardiac biomarkers, though typically without significant coronary artery obstruction.

This case discussed a 66-year-old postmenopausal female who presented with progressive chest discomfort, borderline ST-segment elevation on an electrocardiogram, and mildly elevated cardiac biomarkers, initially raising suspicion for ACS. Urgent cardiac catheterization revealed mild coronary artery disease without significant obstruction, while left ventriculography showed hallmark apical ballooning and preserved basal contractility consistent with TC. Further evaluation revealed an ejection fraction of 24% and grade 2 diastolic dysfunction. Management included guideline-directed medical therapy for heart failure, anticoagulation for thrombus prevention, and comprehensive lifestyle modifications.

This case underscores the diagnostic challenges in distinguishing TC from ACS and highlights the critical role of invasive coronary angiography and advanced imaging. The patient’s presentation was consistent with TC, yet no single acute emotional or physical stressor was identified, suggesting a multifactorial etiology, potentially influenced by chronic hypertension and nicotine use. Postmenopausal women remain at high risk, likely due to hormonal changes affecting myocardial and vascular resilience.

Timely recognition and diagnosis of TC are essential to optimize patient outcomes, as management differs significantly from ACS. This case emphasizes the importance of maintaining a high index of suspicion, particularly in postmenopausal women presenting with ACS-like symptoms, and the value of a multidisciplinary approach to treatment and follow-up.

## Introduction

Takotsubo cardiomyopathy (TC), also known as stress-induced cardiomyopathy or "broken heart syndrome," is a transient cardiac syndrome characterized by acute left ventricular dysfunction that often mimics acute coronary syndrome (ACS). First described in the 1990s, TC typically presents with symptoms of chest pain, shortness of breath, and electrocardiographic abnormalities such as ST-segment elevation, often indistinguishable from those seen in myocardial infarction [1]. However, unlike ACS, TC is usually not associated with significant coronary artery obstruction but instead is thought to be triggered by a sudden surge of catecholamines in response to physical or emotional stressors [2]. This pathophysiological response results in reversible myocardial stunning, which often presents as apical ballooning of the left ventricle with intact basal contractility on imaging studies such as ventriculography or echocardiography [3]. While most patients with TC recover fully with appropriate management, the condition is associated with several acute complications, including heart failure, arrhythmias, and left ventricular thrombus, underscoring the need for appropriate and prompt diagnosis along with thorough monitoring and risk assessment [4].

## Case presentation

**Figure 1:**
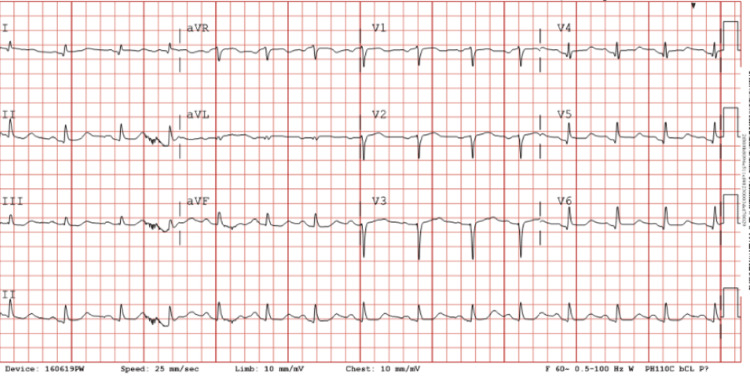
The patient’s EKG significant for borderline ST-segment elevation in the anterolateral leads (V2, V3, V5, V6) with reciprocal ST-segment depression in the inferior leads (V2, V3, AVF) and a prolonged QT interval.

A 66-year-old female with a past medical history of hypertension, hyperlipidemia, chronic back pain, gastroesophageal reflux disease with esophageal stenosis, and nicotine dependence presented with progressive chest discomfort on transfer from another facility. The patient noted that her symptoms started the morning of her admission, and she rated the pain at a 4 out of 10 in intensity. She also reported associated left arm numbness/paresthesia. She denied any recent viral illnesses or significant life stressors. She was a former smoker who quit 17 years ago but continued to use a vaporizer device to consume nicotine. Her relevant family history included unspecified heart disease in her father and coronary artery disease in her paternal uncle, who underwent coronary artery bypass graft surgery. The cardiology team was consulted at that time. She last saw a cardiologist in 2022, where she had a nuclear stress test that revealed no evidence of myocardial ischemia. On admission, she had a blood pressure of 144/92 mmHg, with all other vital signs within normal limits. En route to the hospital, the patient received 162 mg of aspirin, 1 sublingual nitroglycerin, 1 mg of lorazepam, 10 mg of labetalol, and 60 mg of subcutaneous enoxaparin sodium. On physical examination, she had a normal heart rate and rhythm without any murmurs. Her pulmonary effort was normal, with no signs of respiratory distress, and no wheezing, rhonchi, or rales were noted on examination. Her bilateral lower extremities were without edema at that time. A comprehensive metabolic panel was performed on admission that was significant for elevated glucose at 157 mg/dL, low potassium at 3.1 mEq/L, sodium within normal range at 135 mg/dL, calcium within normal range at 8.9 mg/dL, albumin within normal range at 4.1 g/dL, elevated aspartate aminotransferase at 40 IU/L, alanine aminotransferase within normal range at 16 IU/L, alkaline phosphatase within normal range at 82 IU/L, and creatinine within range at 0.47 mg/dL. A complete blood panel was also performed, which was significant for leukocytosis with hemoglobin within normal limits. A chest radiograph was performed on admission that showed no evidence of any significant cardiopulmonary abnormality, and an EKG was performed that showed borderline ST elevation in the anterolateral leads, ST-segment depression in the inferior leads, and a prolonged QT interval with normal sinus rhythm (Figure 1). Her NT-proBNP on admission was 452 pg/mL (reference range 20.0-300.0 pg/mL).

**Figure 2:**
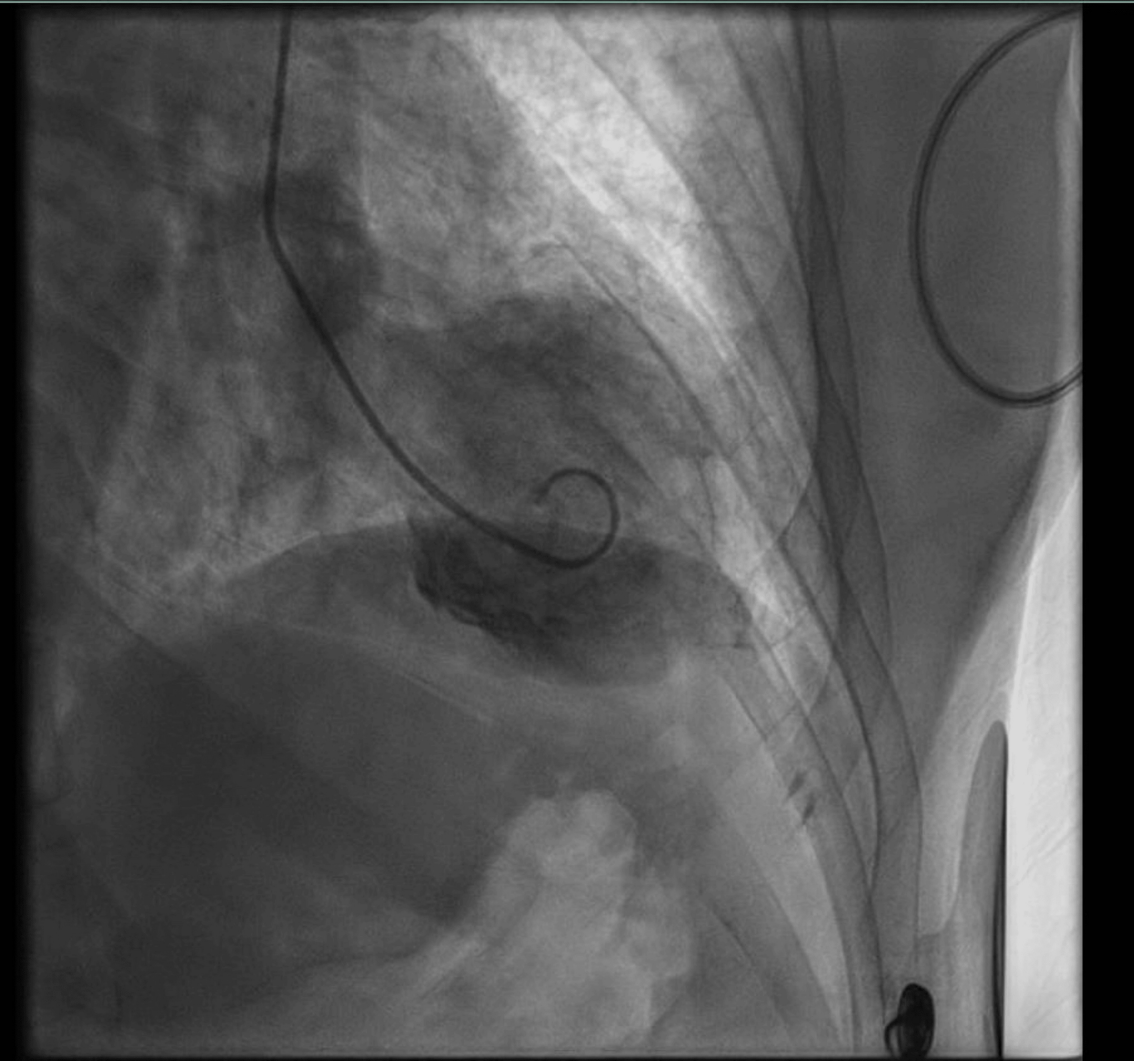
A still image of the patient’s left ventriculogram, showing a basal segment that is contracted with associated dyskinesis of the apex.

After these significant electrocardiogram findings, the patient was transferred to the cardiac catheterization laboratory, where a left heart catheterization and left ventriculogram were performed. On cardiac catheterization, it was discovered that the patient had mild atherosclerotic plaque located in her proximal left anterior descending (LAD) artery and a 30-40% stenosis in her mid-LAD with thrombolysis in myocardial infarction (TIMI) grade 3 flow. In the proximal portion of her left circumflex artery, the patient had a 10-20% stenosis with mild luminal irregularities in her obtuse marginal branches. She also had TIMI 3 flow in this artery. In her right coronary artery, the patient had a stenosis of 20-30% in the mid portion of the artery with associated TIMI 3 flow. On the left ventriculogram, it was discovered that the patient had an ejection fraction of 20%, with preservation of basal contractility and an akinetic distal segment of her left ventricle (Figure 2). These findings were discussed as being the classic appearance of TC. Left ventricular end-diastolic pressure (LVEDP) was 28 mmHg, with no gradient across the aortic valve on a pullback. At this point, it was determined that the patient had severe nonischemic cardiomyopathy, specifically takotsubo type, with a severely elevated LVEDP of 28 mmHg. The recommendation from the interventional cardiology team at this time was to maximize guideline-directed medical therapy for cardiomyopathy. It was also recommended to resume a continuous heparin infusion 2 hours after the procedure with the concern for the development of a left ventricular thrombus in the situation of severe akinesis. 

The patient was then admitted to the medicine floor, where she was planned to start 5 mg of daily apixaban for four weeks, followed by a transition to daily aspirin. The patient had an EKG performed the day after cardiac catheterization that was significant for an ejection fraction of 24%, with LAD territory hypokinesis to akinesis, grade 2 ventricular diastolic dysfunction, a mildly dilated left atrium, mild pulmonary hypertension with a right ventricular systolic pressure of 43 mmHg, and a dilated ascending aorta at 3.50 cm. At this point, the cardiology team cleared the patient for discharge. The patient was discharged on 160 mg of valsartan daily, 12.5 mg of spironolactone daily, 10 mg of empagliflozin daily, 10 mg of ezetimibe daily, and 25 mg of sustained-release metoprolol succinate XL, along with the apixaban as discussed above. On discharge, she was advised to follow up with her primary care physician in one week and reconnect with her primary outpatient cardiologist.

## Discussion

TC, often referred to as stress-induced cardiomyopathy or “broken-heart syndrome,” is a transient cardiac disorder characterized by acute left ventricular dysfunction in the absence of significant coronary artery obstruction. The presentation of TC often mimics ACS, as patients typically experience chest pain and show both electrocardiographic abnormalities and elevated cardiac biomarkers [5]. In this case, borderline ST-segment elevation and prolonged QT interval initially raised suspicion for ACS, prompting urgent cardiac catheterization. However, the absence of significant coronary artery obstruction helped rule out a diagnosis of ACS. This highlights the critical role of invasive coronary angiography in distinguishing TC from ACS.

Additionally, the hallmark feature of TC is basal contractility with akinesis or dyskinesis of the apical segment of the left ventricle [6]. Using advanced imaging techniques, including echocardiography and ventriculography, this ventricular dysfunction is uniquely identified as apical ballooning. This regional wall abnormality differentiates TC from other causes of cardiomyopathy, such as alcohol-induced cardiomyopathy or coronary artery disease, which typically manifest as global, diffuse left ventricular dysfunction [7]. The additional imaging modalities used for this patient were necessary to confirm a diagnosis of TC.

The pathophysiology of TC is thought to involve a catecholamine surge triggered by emotional or physical stress, leading to myocardial stunning and transient left ventricular dysfunction. This excessive adrenergic stimulation results in coronary microvascular dysfunction, myocardial energy depletion via uncoupling of oxidative phosphorylation, and direct cardiomyocyte injury via free radical production, all of which manifest as the characteristic apical ballooning pattern on imaging [8-11]. While emotional stressors, such as the death of a loved one, are well-documented triggers, the temporal relationship between the stressor and the onset of symptoms is crucial. In this patient, the loss of her husband three months before may have contributed to a heightened baseline sympathetic state, but it is unlikely to have directly precipitated her acute event, given the significant time elapsed. It is possible that subclinical or cumulative stressors, compounded by existing cardiovascular risk factors such as hypertension and nicotine use, created a predisposition for TC. 

Treatment for TC is generally supportive and focuses on managing heart failure symptoms and preventing thromboembolic events due to potential left ventricular thrombus formation. Standard management includes using beta-blockers, angiotensin-converting enzyme inhibitors or angiotensin receptor blockers, and statins. In cases of severe left ventricular dysfunction, anticoagulation therapy may also be warranted to reduce thrombus risk [12,13]. Follow-up care is critical to ensure recovery of left ventricular function, with serial echocardiograms recommended to evaluate the resolution of regional wall abnormalities and ejection fraction [14]. Lifestyle modifications, including stress management techniques and addressing cardiovascular risk factors such as hypertension or nicotine use, are also crucial for long-term outcomes. A multidisciplinary approach involving cardiology and primary care is often beneficial to coordinate care and mitigate risk of recurrence. 

TC predominantly affects postmenopausal women, accounting for approximately 90% of cases, with a mean age of onset around 65-70 years [15-17]. The striking prevalence in this demographic is believed to stem from hormonal changes, particularly the loss of estrogen, which plays a cardioprotective role by maintaining coronary blood flow at the microcirculatory level [5]. Overall, TC accounts for an estimated 2% of cases in patients presenting with suspected ACS, though it is likely underdiagnosed due to its variable presentation and overlap with other cardiac conditions [18,19]. Interestingly, despite its classification as a stress-induced condition, not all patients with TC report identifiable triggers, as seen in this case. This highlights the need for greater awareness of atypical presentations and the possibility of underlying subclinical factors contributing to susceptibility. Understanding the epidemiology of TC helps clinicians maintain a high index of suspicion in at-risk populations and avoid misdiagnosis, particularly in postmenopausal women presenting with symptoms resembling ACS.

## Conclusions

This case highlights a classic case of TC, a rare condition mimicking ACS but with distinct characteristics. The patient’s borderline ST-segment elevation and prolonged QT interval raised initial concerns for ACS, prompting cardiac catheterization to exclude significant coronary artery obstruction. Ventriculography confirmed hallmark apical ballooning and basal contractility, guiding management. Though the exact trigger was unclear, the patient’s hypertension, nicotine use, and possible stressors likely contributed, emphasizing the importance of keeping a broad but grounded differential diagnosis and considering TC, especially in postmenopausal women, who are disproportionately affected.

Management of TC involves optimizing heart failure treatment, mitigating thromboembolic risks, addressing cardiovascular risk factors, and educating patients on stress management and lifestyle changes. Regular follow-up is crucial to monitor left ventricular recovery and prevent recurrence. This case underscores the need for clinicians to maintain a high index of suspicion for TC in ACS-like presentations without significant coronary artery disease. Early recognition and comprehensive care can improve outcomes and enhance the quality of life for patients with this unique syndrome. 
